# METABOLIC AND NUTRITIONAL REPERCUSSIONS OF LIVER DISEASE ON CHILDREN: HOW TO MINIMIZE THEM?

**DOI:** 10.1590/1984-0462/2022/40/2020149

**Published:** 2021-05-26

**Authors:** Beatriz Polisel Mazzoni, Bruna Voltani Lessa, Patricia Zamberlan

**Affiliations:** a Universidade de São Paulo, São Paulo, SP, Brazil.; b Hospital Municipal Infantil Menino Jesus, São Paulo, SP, Brazil.

**Keywords:** Biliary atresia, Child, Nutritional status, Nutrition therapy, Liver transplantation, Atresia biliar, Criança, Estado nutricional, Terapia nutricional, Transplante de fígado

## Abstract

**Objective:**

To describe the metabolic and nutritional repercussions of chronic liver disease (CLD), proposing strategies that optimize nutritional therapy in the pre- and post-liver transplantation (LT) period, in order to promote favorable clinical outcomes and adequate growth and development, respectively.

**Data sources:**

Bibliographic search in the PubMed, Lilacs and SciELO databases of the last 12 years, in English and Portuguese; target population: children from early childhood to adolescence; keywords in Portuguese and their correlates in English: “Liver Transplant,” “Biliary Atresia,” “Nutrition Therapy,” “Nutritional Status,” and “Child”; in addition to Boolean logics “and” and “or,” and the manual search of articles.

**Data synthesis:**

Malnutrition in children with CLD is a very common condition and an important risk factor for morbidity and mortality. There is an increase in energy and protein demand, as well as difficulties in the absorption of carbohydrates, lipids and micronutrients such as fat-soluble vitamins and some minerals. An increase in the supply of energy, carbohydrates and proteins and micronutrients, especially fat-soluble vitamins, iron, zinc and calcium, is suggested, except in cases of hepatic encephalopathy (this restriction is indicated for a short period).

**Conclusions:**

Based on metabolic changes and anthropometric and body composition monitoring, a treatment plan should be developed, following the nutritional recommendations available, in order to minimize the negative impact of malnutrition on clinical outcomes during and after LT.

## RESUMO

**Objetivo:**

Revisar de forma descritiva as repercussões metabólicas e nutricionais da doença hepática crônica, propondo estratégias que aperfeiçoem a terapia nutricional nos períodos pré e pós transplante hepático (TxH), para promover desfechos clínicos favoráveis e crescimento e desenvolvimento adequados, respectivamente.

**Fontes de dados:**

Pesquisa bibliográfica nas bases de dados PubMed, Lilacs e SciELO dos últimos 30 anos em língua inglesa e portuguesa; população-alvo: crianças da primeira infância até a adolescência; palavras-chave em português e seus correlatos em inglês: “transplante de fígado”, “atresia biliar,” “terapia nutricional”, “estado nutricional” e “criança”; além das lógicas booleanas *and* e *or* e da busca manual de artigos.

**Síntese dos dados:**

A subnutrição em crianças com doença hepática crônica é uma condição muito comum e um importante fator de risco para a morbimortalidade. Ocorre aumento das demandas de energia e proteínas, bem como dificuldades na absorção de carboidratos, lipídeos e de micronutrientes como vitaminas lipossolúveis e alguns minerais. Sugere-se incremento no aporte de energia, carboidratos, proteínas e micronutrientes, sobretudo de vitaminas lipossolúveis, ferro, zinco e cálcio, exceto em casos de encefalopatia hepática (a restrição é indicada por um curto período).

**Conclusões:**

Com base nas alterações metabólicas, no monitoramento antropométrico e na composição corporal, um plano terapêutico deve ser elaborado, seguindo as recomendações nutricionais disponíveis, com o objetivo de minimizar o impacto negativo da subnutrição nos desfechos clínicos durante e após o transplante hepático.

## INTRODUCTION

Numerous chronic liver diseases (CLD) caused by infections, anatomical, genetic, or metabolic alterations can manifest in the pediatric age group.^1^ ^,^ ^2^ The most common cause in infants in Brazil is biliary atresia, characterized by partial and/or complete obstruction of the extrahepatic bile ducts and the main indication for liver transplantation (LT), a treatment increasingly adopted in pediatric patients in both developed and developing countries.^2^ ^,^ ^3^ ^,^ ^4^ ^,^ ^5^ ^,^ ^6^ ^,^ ^7^

In CLD, as well as in biliary atresia, liver dysfunction is responsible for changes in the metabolism of carbohydrates, lipids, proteins, and vitamins and in their intestinal absorption. In addition to the low caloric and protein intake due to gastric compression associated with ascites or hepatosplenomegaly, there is an increase in proinflammatory cytokines, which promote a hypermetabolic state, negatively impacting patients’ clinical conditions and prognosis.^8^ ^,^ ^9^ ^,^ ^10^ ^,^ ^11^ ^,^ ^12^

When cholestasis is present, the nutritional consequences are more severe, as the absence of bile in the intestine can cause steatorrhea, directly related to a malabsorption of lipids and consequent deficiency of fat-soluble vitamins and minerals, which greatly aggravates the patients’ nutritional status.^2^ ^,^ ^13^

Therefore, malnutrition in patients with CLD is a well-established condition, present in 60 to 80% of children in the terminal stage of the disease,^14^ ^,^ ^15^ ^,^ ^16^ and which negatively impacts clinical outcomes in the post-LT period.^12^ ^,^ ^17^ Taking this into consideration, the medical and nutritional performance in CLD is fundamental and aims at adequately maintaining the patients’ nutritional condition, enabling better clinical outcomes during treatment.

The objective of this study is to descriptively review the metabolic and nutritional repercussions of CLD, proposing strategies that optimize nutritional therapy in the pre- and post-LT periods, in order to promote favorable clinical outcomes and adequate growth and development, respectively.

## METHOD

The research was carried out by two independent reviewers, in the PubMed, Latin American & Caribbean Health Sciences Literature (Lilacs), and Electronic Scientific Library Online (SciELO) databases, using the following keywords in Portuguese and English: *liver transplantation*, *biliary atresia*, *nutrition therapy*, *nutritional status,* and *child*, and also using the Boolean logics *and* and *or*. The filters used for the preparation of the LT nutritional care proposal were: articles from the last 12 years published in English and Portuguese and the target population of children from early childhood to adolescence. The survey was conducted from May to October 2019, and articles of the following types were excluded from the survey: editorials, letters, comments, and case studies. Thus, 312 articles were found.

**Figure 1:**
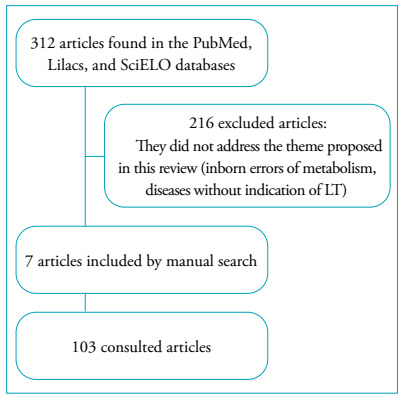
Flowchart of article selection.

Subsequently, articles were selected based on titles and abstracts, excluding those not related to the proposed theme - cholestatic liver diseases, liver transplantation, and nutritional assessment -, which resulted in 96 articles. Then, the articles were completely read, and seven more articles were included (used in the initial studies) through manual search. The flowchart of article selection is shown in Figure 1.

## RESULTS

### Energy requirement

Malnutrition represented by the loss of muscle mass and fat reserves, as well as liver dysfunctions, are directly related to the increase in basal energy expenditure, which characterizes a hypermetabolic state. By using indirect calorimetry, according to Pierro et al.,^18^ children with biliary atresia may have a 30 to 40% increase in their needs. In addition to other factors, such as gastric compression, insufficient food intake, anorexia, and malabsorption of nutrients, such changes may explain the low weight and height of these patients.^9^ ^,^ ^12^ ^,^ ^19^ ^,^ ^20^

Current recommendations for patients with cholestasis suggest a nutritional supply of approximately 130% of energy requirements according to age, which may exceed 150% in the presence of liver cirrhosis.^7^ ^,^ ^19^ ^,^ ^21^ ^,^ ^22^ Taking this into consideration, it is recommended to use indirect calorimetry to estimate energy requirements as the gold standard. In its absence, prediction equations validated in pediatrics can be used, considering the additions previously described and the patients’ clinical evolution.^23^

### Carbohydrates

Liver diseases are characterized by depletion of hepatic glycogen, changes in insulin synthesis, and reduced signaling in the liver. Failure of these homeostatic mechanisms can result in hypoglycemia, hyperglycemia, or glucose intolerance.^9^

Hypoglycemia occurs due to limited glycogen stores and the inability to mobilize it, especially after prolonged periods of fasting. Special attention should be payed to infants because they have smaller glycogen stores and, consequently, a higher risk of hypoglycemia.^14^ ^,^ ^24^ ^,^ ^25^

 Hyperglycemia, secondary to insulin resistance and hypertriglyceridemia, may indicate decreased glucose tolerance caused by CLD. Furthermore, these metabolic changes are suggestive of a possible imbalance in the supply of carbohydrates in the diet, which must always be adjusted and balanced.^16^ ^,^ ^21^ ^,^ ^23^ ^,^ ^24^

Hence, it is recommended to maintain carbohydrates between 40 and 60% of the total energy requirements, considering the presented symptoms and the child’s age group.^20^ ^,^ ^24^

### Proteins

Depending on the degree of liver function impairment, there may be a decrease in the production of plasma proteins such as albumin, prealbumin, transferrin, retinol-binding protein, lipoproteins, and clotting factors. The mechanisms responsible for these changes are not yet fully elucidated, but they may be linked to increased protein catabolism or reduced protein synthesis.^24^ ^,^ ^26^

The increase in protein requirements may also be related to the storage of glycogen in the impaired liver and the decrease in glycogenolysis, which stimulates neoglycogenesis, diverting the use of amino acids for energy production.^23^ ^,^ ^27^ Thus, muscle mass depletion implies a reduction in the plasma concentration of branched-chain amino acids (BCAAs), an increase in aromatic amino acids (phenylalanine, tyrosine, tryptophan) and, consequently, the formation of a negative nitrogen balance.^26^ ^,^ ^27^ ^,^ ^28^ ^,^ ^29^

Studies by Mager et al.^27^ and Chin et al.^28^ demonstrated a significant improvement in the nitrogen balance of children with CLD supplemented with BCAAs, suggesting that the depletion of muscle mass in advanced liver disease results in an increase in the requirements of BCAAs. Nel and Terblanche^14^ also reported that BCAAs supplementation improved muscle mass gain and nitrogen balance. However, to date, no BCAAs supplementation doses have been established for the pediatric population. In addition, formulas enriched with these amino acids are not very accessible given the price and availability in the market.

Therefore, a protein supply of 130 to 150% of the age recommendations for healthy children is recommended for children and adolescents with CLD in the presence of cholestasis.^24^ According to Nel and Terblanche,^14^ protein intake of 3 to 4g/kg of body weight per day can be considered, depending on the biochemical tests and the patients’ nutritional status.

Although moderate elevations of ammonia are frequently present due to the impossibility of their conversion to urea by the liver and protein catabolism, it is suggested the prolonged non-restriction of proteins in the absence of hepatic encephalopathy, and a protein intake of at least 2g/kg/day is recommended.^23^ Protein restriction, in an attempt to prevent the development of hepatic encephalopathy in children, is less appropriate, considering the increased demand for proteins for growth. Charlton et al.^29^ demonstrated in their study that the intake of up to 4g/kg/day of proteins does not precipitate hepatic encephalopathy in children with severe CLD.^10^ ^,^ ^30^

In the same study of Charlton et al.,^29^ in which ten children with advanced cirrhosis and malnutrition received enteral nutritional therapy via the gastric route for eight weeks, composed of whey protein, fat (34% of medium-chain triglycerides [MCT] and 66% long-chain triglycerides [LCT]), and glucose polymers, the researchers showed that nutritional rehabilitation using 130% of the recommended caloric intake and 4g/kg/day of protein improved nutritional status without signs of hyperammonemia. Therefore, adequate protein intake (2-4g/kg/day) should be stimulated and recommended.^10^ ^,^ ^24^ ^,^ ^26^ ^,^ ^30^

Protein restriction (<2g/kg/day) can be only considered during episodes of severe encephalopathy, though not exceeding the three-day limit, according to current recommendations.^24^ ^,^ ^31^ Upon positive evolution of the hepatic encephalopathy clinical condition, the adequate protein supply must be resumed, with the objective of attenuating protein catabolism and the worsening of nutritional status, especially in the pre-transplantation period, and not impairing the age-appropriate growth and development .^18^ ^,^ ^23^ ^,^ ^24^ ^,^ ^26^ ^,^ ^30^ ^,^ ^31^

### Lipids

There is an imbalance between the lipolytic and lipogenic pathways in liver failure. In most cases, lipolysis is increased and the syntheses of triglycerides, phospholipids, lipoproteins, and bile salts are reduced. In cholestatic liver diseases, reduced bile flow impairs fat absorption and can cause steatorrhea, which is also favored by decreased carnitine synthesis, which is essential for the transport of LCT into the hepatocytes.^10^ ^,^ ^23^ In this scenario, LCT digestion and absorption are more impaired than MCT digestion and absorption, as they are more soluble in water and are readily absorbed by enterocytes in the absence of micelles. The absence of bile salts in the intestinal lumen makes it difficult to absorb essential fatty acids (linoleic and linolenic acids) and fat-soluble vitamins (A, D, E, and K).^10^ ^,^ ^23^ ^,^ ^24^

Therefore, the diet of patients with CLD can be supplemented with MCT, not exceeding 80% of the total fat intake, considering that the lack of LCT can lead to deficiencies of essential fatty acids, important for normal growth and cerebral development. These essential fatty acids are easily found in egg yolks and vegetable oils, for example.^10^ ^,^ ^14^ Nevertheless, the fat requirements of patients with CLD will depend, as well as other macronutrients, on the nutritional status and on the presence and severity of lipid malabsorption, which is evidenced by the loss of fat in feces and the reduction in measurements of tricipital skinfold.^24^

In clinical practice, patients generally have normolipidic diets, respecting the recommendations of 25 to 30% of the total energy value, as there are no specific recommendations for this population.^24^ According to Sultan et al.,^30^ most infant formulas contain insufficient amounts of MCT, but provide a good energy balance for newborns (NB) and cholestatic infants in terms of MCT/LCT ratio. The authors also emphasize that the lipids supplied to this population must respect the ratio of 30 to 60% of MCT, with at least 40% of LCT to prevent the deficiency of essential fatty acids (EFA) and favor the adequate child development. Current recommendations suggest that the MCT/LCT ratio should be initially offered at a ratio of 30/70%.^24^

Hence, formulas enriched with MCT can be indicated to infants, as well as the addition of a total daily dose of 1 to 2mL/kg/day of MCT to the main meals of cholestatic children.^15^ ^,^ ^23^ ^,^ ^24^ ^,^ ^30^ The low palatability of this oil can make food unpleasant and, consequently, reduce food intake. Therefore, the benefit of adding MCT should be weighed, considering the patients’ acceptance and nutritional status.

### Fat-soluble vitamins

Due to impaired metabolism of macronutrients, especially lipids, deficiency of fat-soluble vitamins (A, E, D, and K) may be present in 20 to 35% of patients with cholestatic liver disease, especially in those with low intake of vitamins-source foods and who do not receive supplementation. These deficiencies result in visual and skin changes, neurological disorders, rickets, osteoporosis, cerebellar ataxia, and coagulopathy.^23^ ^,^ ^24^ ^,^ ^31^ ^,^ ^32^ ^,^ ^33^ ^,^ ^34^

Shneider et al.^35^ demonstrated that the overall prevalence of deficiency of fat-soluble vitamins in pediatric patients with biliary atresia, six months after undergoing the Kasai procedure, varied from 10 to 37%. On the other hand, when only considering patients with total bilirubin greater than 2mg, this prevalence percentage considerably increased, reaching 46% vitamin K, 50% vitamin E, 79% vitamin D, and 100% vitamin A, which reinforces the need for supplementation in this population.

Such deficiencies are mainly related to fat malabsorption and, therefore, most patients with CLD can receive a standard, age-appropriate multivitamin dose, based on laboratory results, according to Leon and Lerret.^31^ In addition, current recommendations also suggest replacement of fat-soluble vitamins in water-soluble preparations, facilitating their absorption by cholestatic patients.^24^ ^,^ ^36^ ^,^ ^37^ However, unfortunately, such preparations are not available in most pediatric hepatology centers in our country.

### Minerals

The most commonly observed deficiencies of minerals and trace minerals are: iron, zinc, and calcium, which makes the monitoring of these micronutrients essential for adopting any type of supplementation.^30^

Iron and zinc levels should be periodically monitored, considering that in patients with cholestasis, gastrointestinal bleeding and excessive losses of zinc in feces and urine can lead to deficiency.^20^ Iron deficiency also occurs in children with portal hypertension and zinc deficiency should be investigated in children with abnormal growth or who have clinical signs such as skin rashes and diarrhea.^38^ In a study conducted by Mattar et al.,^39^ higher levels of ferritin and lower iron intestinal absorption were observed in patients with cholestasis compared with patients with anemia and without cholestasis. These findings presuppose a relationship between anemia and inflammation in cholestasis, and oral iron therapy is suggested. However, it should be noted that iron supplementation must be treated with caution, considering its association with increased oxidative stress and fibrogenesis in patients with CLD, which can lead to hepatotoxicity.^20^ ^,^ ^39^

In turn, zinc in circulation is predominantly bound to albumin; in conditions of severe malnutrition, inflammation and CLD, there may be a reduction in its circulating level. In addition, zinc is a necessary element for the synthesis of alkaline phosphatase, whose reduced values may suggest deficiency. However, alkaline phosphatase must be considered with other parameters, as patients with cholestasis and/or bone disease naturally present high levels.^24^ ^,^ ^40^ If zinc deficiency is present, supplementation of 1mg/kg/day of elemental zinc, such as zinc sulfate, is recommended.^23^

Malabsorption of fat is also associated with lower intestinal absorption of calcium and phosphate, which can favor the development of bone diseases, which in turn do not necessarily respond to normalization of the serum level of vitamin D.^30^ Serum calcium is regulated by vitamin D, parathyroid hormone, and calcium intake. Approximately 50% of this mineral is in ionized form in the circulation, whereas the rest is in inactive form, linked to albumin and serum globulins.^23^ Therefore, disorders associated with the decrease in serum albumin are related to the reduction in total serum calcium, but do not alter the ionized form. Hence, ionized calcium should be used as a reference in patients with hypoalbuminemia.^23^ ^,^ ^24^

During vitamin D replacement therapy, for example, all children should be supplemented with calcium, regardless of the presence or absence of hypocalcemia, so as to avoid deficiency. In addition, calcium replacement can assist in the child’s bone growth and mineralization process, an extremely important factor in this age group.^24^

Regarding sodium, the current recommendations^24^ suggest control in cases of ascites or fluid overload, and the amount of 1 to 2mEq kg^-1^/day^-1^ should be offered.

As for other minerals, Chin et al.^28^ documented in their study low general levels of micronutrients in children with cholestasis. Selenium and magnesium deficiencies, for instance, can also be found in these children, and should be supplemented according to the guidance on plasma levels.^26^

In summary, it is worth emphasizing the importance of monitoring laboratory tests, clinical symptoms and signs, and nutritional recommendations - Dietary Reference Intakes (DRI), Recommended Dietary Allowance (RDA), and Tolerable Upper Intake (UL) levels - for age, in such a way that an appropriate nutritional therapy is established for assisting in the achievement of favorable clinical outcomes.^23^ ^,^ ^24^

### Nutritional assessment

Considering that malnutrition is associated with increased morbidity and mortality, such as increased risk of infections, reduced intestinal function, and longer hospital stay, the assessment of nutritional status becomes essential in identifying patients who are at nutritional risk, and should be performed at the first nutritional consultation and subsequent appointments.^12^ ^,^ ^14^ ^,^ ^41^

The nutritionist must pay attention to the details of the patients’ clinical and nutritional history, in order to better guide those responsible and propose effective interventions in order to prevent or correct deficiencies, optimize growth/development, and reduce morbidity and mortality.^10^ ^,^ ^42^

Regarding anthropometry, the main pillar of nutritional assessment in pediatrics, such is influenced by changes in body composition in children with CLD. Overall, the measures of weight-for-age and weight-for-height underestimate the degree of malnutrition of these patients due to ascites, hepatosplenomegaly, and fluid retention. In this context, arm circumference (AC) and tricipital skinfold are the most suitable assessment parameters, considering that they better discriminate the patients’ nutritional status, as they are less influenced by edema and more sensitive to changes that occur in a short time interval, when compared with weight and height measurements.^14^ ^,^ ^12^ ^,^ ^19^ ^,^ ^20^

In the service of the authors of the present study, data not yet published showed that, of the 47 patients submitted to LT in the last year, 36.2% had malnutrition by the percentiles of arm circumference-for-age - AC (p <5). Zamberlan et al.^12^ found 50% of malnutrition according to height-for-age and 61.6% according to arm circumference-for-age. Furthermore, they observed a negative correlation between Z-score values for arm circumference-for-age and length of stay in days. Recent data from the United States of America showed that children with terminal liver disease had, on average, a 23% reduction in muscle mass and an increase of 69% in visceral fat, and of 29% in subcutaneous fat compared with healthy children.^43^ For this reason, in addition to measures of weight and height, it has been suggested in the assessment of these patients to estimate measures indicative of body composition, such as AC and tricipital skinfold, in such a way that they can more accurately classify their nutritional status and, thus, establish an effective nutritional therapeutic plan.^12^ ^,^ ^20^ ^,^ ^44^

### Nutritional recommendations

Based on the metabolic and nutritional repercussions discussed in this study, Table 1 summarizes the nutritional recommendations found in the literature for the nutritional management of children and adolescents with cholestatic CLD, especially during the period preceding the LT, for proportional favorable post-procedure outcomes. It is noteworthy that nutritional therapy, whether enteral or parenteral, should always be considered when the intake of macro- and micronutrients is inadequate or the oral diet is unable to provide daily nutritional goals, even though there is the option of using modules based on carbohydrates, proteins, and fats in the diet. It is up to the nutritionist to monitor the nutritional evolution of the patients, to assess their needs and, together with the team, to decide the best food route.^24^ ^,^ ^45^

**Table 1:** Nutritional recommendations for children with chronic cholestatic liver disease in the pre-liver transplantation period.

Energy/nutrients	Recommendations
Energy	130 to 150% of recommendations according to age*
Carbohydrate	40 to 60% of TEV
Protein	3 to 4g/kg of weight/day Cholestasis: 130 to 150% of recommendations according to age Restriction (<2g/kg/day) only in cases of hepatic encephalopathy, for three consecutive days at most.
Lipids	40 to 60% of TEV MCT/LCT 30/70% (minimum 40% LCT) Infants: 1 to 2mL/kg/day of MCT
Vitamin A	5,000 IU/day children <10 kg** 10,000 IU/day children >10 kg**
Vitamin D	2,000-5,000 IU/day**
Vitamin E	15-25 IU kg/day**
Vitamin K	2-5 mg/day**
Iron Zinc Calcium Selenium Magnesium	Performance of individual assessment and, if necessary, recommendation of supplementation according to the RDAs for age without exceeding the UL.
Sodium	Restriction in cases of ascites or fluid overload

*Based on prediction equations validated for pediatrics. **Individual assessment of the need for supplementation. RDA: Recommended Dietary Allowances; MCT: medium-chain triglycerides; LCT: long-chain triglycerides; UL: Tolerable Upper Intake; IU: international unity; TEV: total energy value.

Children with cholestatic CLD have a high nutritional risk and malnutrition, which represents one of the complications of great impact on their survival. Metabolic changes that occur in these patients include increased energy and protein requirements, changes in glucose homeostasis, difficulty in absorbing lipids and micronutrients, such as fat-soluble vitamins and some minerals, which have a negative impact on their nutritional condition, consequently determining unfavorable clinical outcomes in the perioperative period.

Based on metabolic changes and anthropometric and body composition evaluations, a treatment plan should be developed, following the nutritional recommendations available, in order to minimize the negative impact of malnutrition on clinical outcomes during and after LT.
